# Other PHAC publications

**DOI:** 10.24095/hpcdp.45.7/8.04

**Published:** 2025-08

**Researchers from the Public Health Agency of Canada also contribute to work published in other journals and books. Look for the following articles published in 2025:**

Afifi TO, McCarthy JA, Osorio A, MacGowan L, Taillieu TL, Stewart-Tufescu A, […] **Tonmyr L**, et al. Child abuse prevalence estimates in Canada; comparisons of nationally representative data from 2012 to 2022: a population-based study. Lancet Reg Health Am. 2025;45:101072. https://doi.org/10.1016/j.lana.2025.101072

**Baysac D-J**, Guay M, **Chen R**, Dub , MacDonald SE, Driedger SM, **Gilbert NL**. Did inequalities in COVID-19 vaccination resolve over time? Insights from the Canadian Community Health Survey. Vaccine. 2025;56:127153. https://doi.org/10.1016/j.vaccine.2025.127153

**Capaldi CA, Wassef K, Varin M, Vallires E, Roberts KC.** Sense of control and positive mental health outcomes among adults in Canada during the COVID-19 pandemic. Health Rep. 2025;36(4):14-26. https://doi.org/10.25318/82-003-x202500400002-eng

Chaput J-P, Tremblay MS, Goldfield GS, **Prince SA**, Biswas A, Colley RC, **Lang JJ**. Is working from home good for mental health and well-being? Associations between work location, self-rated mental health, life satisfaction, and life and work stress among Canadian adults. Ment Health Prev. 2025;38:200418. https://doi.org/10.1016/j.mhp.2025.200418

**Cheta N, Zakaria D, Demers A, Abdullah P, Aziz S.** Association between pre-existing chronic conditions and severity of first SARS-CoV-2 infection symptoms among adults living in Canada: a population-based survey analysis from January 2020 to August 2022. BMC Public Health. 2025;25(1):981. https://doi.org/10.1186/s12889-025-22041-7

Davenport MH, Ruchat SM, **Jaramillo Garcia AP**, Ali MU, Forte M, Beamish N, et al. 2025 Canadian guideline for physical activity, sedentary behaviour and sleep throughout the first year post partum. Br J Sports Med. 2025;59(8):515-26. https://doi.org/10.1136/bjsports-2025-109785

**Demchenko I, Prince SA**, Merucci K, Cadenas-Sanchez C, Chaput JP, Fraser BJ, […] **Lang JJ**. Cardiorespiratory fitness and health in children and adolescents: an overview of systematic reviews with meta-analyses representing over 125 000 observations covering 33health-related outcomes. Br J Sports Med. 2025. https://doi.org/10.1136/bjsports-2024-109184

**De Rubeis V, Tonmyr L, Tanaka M**, Afifi T, Catherine N, Osorio A, et al. The psychometric properties of childhood physical and sexual abuse measures in two Canadian samples of youth and emerging adults. PLoS ONE. 2025;20(5):e0318448. https://doi.org/10.1371/journal.pone.0318448

Ding X, McVarnock A, Li M, Coplan RJ, **Ooi LL**, Yu J, et al. Motivations for social withdrawal and socio-emotional functioning among urban/suburban Chinese children. J Appl Dev Psychol. 2025;98:101787. https://doi.org/10.1016/j.appdev.2025.101787

**Ghanem S, Marulappa N, Qiang V**. Key considerations for applying intersectionality theory to partner and stakeholder engagement in public health. Can J Public Health. 2025. https://doi.org/10.17269/s41997-025-01023-7

**Ghanem S, Moraleja M, Gravesande D, Rooney J**. Integrating health equity in artificial intelligence for public health in Canada: a rapid narrative review. Front Public Health. 2025;13:1524616. https://doi.org/10.3389/fpubh.2025.1524616

Halsall T, Mahmoud K, Drabenstott M, **Orpana H**, Iyer SN, Kristjansson A, et al. Processes of development related with the implementation of the Icelandic prevention model in a rural Canadian community. Discov Public Health. 2025;22(1):67. https://doi.org/10.1186/s12982-025-00443-7

Kobewka D, Hakimjavadi R, Yin CY, **Scott M**, Talarico R, Ramsay T, et al. Cognitive and functional decline among long-term care residents. JAMA Netw Open. 2025;8(4):e255635. https://doi.org/10.1001/jamanetworkopen.2025.5635

Lm S, **Raza S, Hansen L**. Engaging scientists in science policy: experiences from Canada. Public Health Rev. 2025;46:1607130. https://doi.org/10.3389/phrs.2025.1607130

**Liu L, Contreras G, Thompson W**. Repeat self-harm hospitalizations in Canada: a survival analysis. Inj Epidemiol. 2025;12(1):26. https://doi.org/10.1186/s40621-025-00576-y

**McKenzie K, Belanger B, Parshad S, Xie L, Grywacheski V**, Fidler-Benaoudia M. Late mortality among survivors of childhood cancer in Canada: a retrospective cohort study. Pediatr Blood Cancer. 2025;72(7):e31700. https://doi.org/10.1002/pbc.31700

**Prince SA, Doan N, Butler GP, Srugo SA**, Winters M, Colley RC, […] **Lang JJ**. Cycling infrastructure and transportational and recreational physical activity in Canadians. J Transp Health. 2025;42:102046. https://doi.org/10.1016/j.jth.2025.102046

**Rabeenthira P**, Zagrodney KAP, King EC, Nichol KA, McKay SM. What drove clients’ decisions to pause personal homecare services before and during the pandemic? Health Serv Insights. 2025;18. https://doi.org/10.1177/11786329251335877

**Rotondo J, VanSteelandt A**, Kouyoumdjian F, Bowes MJ, **Kakkar T**, Jones G, […] **Murray R, Schleihauf E**, […] **Jackson B**, […] Shah D, Rees EE. Substance-related acute toxicity deaths in Canada from 2016 to 2017: protocol for a retrospective chart review study of coroner and medical examiner files. JMIR Public Health Surveill. 2025;11:e49981. https://doi.org/10.2196/49981

**Sterian M, Naganathan T, Corrin T, Waddell LA**. Evidence on the associations and safety of COVID-19 vaccination and post COVID-19 condition: an updated living systematic review. Epidemiol Infect. 2025;153:e62. https://doi.org/10.1017/S0950268825000378

**Turner SE, Lang JJ, Doan N, Varin M, Thompson W, Dopko RL**. Examining interactions between chronic pain, positive mental health and coping on past-year suicidal ideation in a Canadian sample. SSM Ment Health. 2025;7:100450. https://doi.org/10.1016/j.ssmmh.2025.100450

**Wang JM**, Ng E, Kohen D, Viau R, Rank C, Grundy A. All-cause and cause-specific hospitalization rates among temporary and permanent residents living in Canada: a linkage study. Can J Public Health. 2025. https://doi.org/10.17269/s41997-025-00996-9

